# Trypanocidal Treatment for Chronic Chagas Disease: Past, Present, and Future

**DOI:** 10.1590/0037-8682-0242-2025

**Published:** 2025-10-27

**Authors:** Alejandro Marcel Hasslocher-Moreno

**Affiliations:** 1 Instituto Nacional de Infectologia Evandro Chagas, Fundação Oswaldo Cruz, Rio de Janeiro, RJ, Brazil.

**Keywords:** Chagas disease, Trypanocidal treatment, Adverse drug reaction, Guidelines, Benznidazole, Nifurtimox

## Abstract

This article reviews the trypanocidal therapy for chronic Chagas disease, emphasizing its indications, efficacy, limitations, and future perspectives. The etiological treatment is based on the use of benznidazole and nifurtimox, both of which were developed over five decades ago. These drugs are most effective in the acute phase, but are also recommended for children, adolescents, and adults aged <50 years without severe organ damage, and women of childbearing age to prevent congenital transmission. Adherence to treatment is limited by adverse drug reactions, which affect approximately half of the patients, leading to treatment discontinuation in approximately 30% of cases. The cure criteria included parasitological, serological, and clinical responses that required long-term follow-up. Clinical trials and systematic reviews have shown heterogeneous results that are influenced by age, clinical stage, and geographical region. Recent public policies supported by non-governmental organizations and academic networks have expanded access to diagnosis and treatment, although structural and informational barriers persist. New therapeutic strategies include shortened benznidazole regimens, drug repositioning, and combination therapies aimed at reducing adverse drug reactions and improving efficacy. Novel molecules with distinct mechanisms and vaccines with therapeutic and preventive potentials are under investigation. Despite these advances, the challenge remains in translating these innovations into concrete benefits for affected populations, particularly in the most vulnerable regions.

## INTRODUCTION

More than a century after the discovery of Chagas disease (CD), safe, efficient, and effective drugs against all clinical forms of the disease are lacking. Etiological treatment remains limited to two drugs, nifurtimox (NFX) and benznidazole (BZN), which were developed in the mid-1960s and the early 1970s, respectively^1^. These drugs are mainly indicated for acute, reactivated, or congenital cases; recent chronic infections in children and adolescents; and women of childbearing age due to their indirect impact on congenital transmission. Although robust evidence is lacking, treatment is also recommended for the indeterminate form in adults up to 50 years of age^2^. The use of NFX and BZN is associated with a high incidence of adverse drug reactions (ADRs), leading to treatment discontinuation within the first week in approximately 20% of patients. Thus, clinical management of ADRs presents a challenge for physicians and discourages patients from completing therapy^3^.

Despite the lack of precise data on the number of individuals affected by CD, it is estimated that millions of people live with chronic CD (CCD) without access to diagnosis and, consequently, cannot benefit from trypanocidal therapy. Recently, public health policies have supported the identification and treatment of individuals with CCD^4^. Perspectives on the etiological treatment of CD include changes in the dosage of available drugs to improve patient adherence, drug repositioning, and BZN-based combination therapy to reduce ADRs, and the development of new drugs with novel therapeutic targets and vaccines^5^.

## HISTORICAL BACKGROUND

Three years after the discovery of CD, the first in vivo study was conducted to identify drugs with trypanocidal activities. Compounds, such as arsenic, fuchsin, tartar emetics, and mercury, have shown no favorable results^6^. In a review on CD, Chagas confirmed the absence of effective medications for the disease^7^. Between the 1940s and the 1950s, various drugs already in clinical use, such as antibiotics, antifungals, and antimalarials, were tested^8^. However, treatment was only achieved in patients with both acute and chronic cases through consistent experimental treatment with nitrofuran compounds, yielding parasitological cure rates of 50% for acute cases and 20%-0% for chronic cases^9^ ^-^ ^13^. It was not until the 1960s and 1970s that drugs with trypanocidal activity, such as NFX and BZN, were developed^14^ ^,^ ^15^. With significant results in the acute phase, recent chronic infections, and congenital infection^16^ ^,^ ^17^, these two drugs have become the cornerstone of therapeutic trials in the late chronic phase of CD^18^ ^-^ ^21^. In the 1980s, drugs used to treat other diseases, particularly ketoconazole and allopurinol, were tested^22^. However, subsequent clinical evaluations showed that these drugs do not have the same efficacy as NFX and BZN^23^ ^,^ ^24^.

Following studies have further advanced the understanding of the benefits of trypanocidal treatment, demonstrating positive effects on parasitemia reduction, serological response, and cardiomyopathy progression^25^ ^-^ ^27^. Randomized studies of BZN have broadened our understanding of its efficacy in the chronic phase^28^ ^-^ ^30^. However, the indication of etiological treatment for CCD remains controversial, mainly because of challenges in evaluating its efficacy. In the early 2000s, prospective studies on BZN for CCD provided the first evidence supporting the clinical efficacy of its trypanocidal effects^31^ ^-^ ^34^. Since 2010, more robust randomized clinical trials have been conducted, including CHAGASAZOL^35^, BENEFIT^36^, STOP CHAGAS^37^, E1224^38^, MULTIBENZ^39^, BENDITA^40^, and TESEO^41^, further supporting the trypanocidal action of BZN.

## TREATMENT INDICATIONS

Until the 1980s, indications for the etiological treatment of CCD lacked a consensus. The prevailing belief is that autoimmunity is the main mechanism underlying both cardiac and digestive clinical manifestations of the disease^42^. Consequently, the role of *Trypanosoma cruzi* was considered marginal; therefore, treating patients with trypanocidal drugs would have little to no impact on the course of CCD. Studies began to emerge only in the 1990s, showing evidence that etiological treatment has a positive influence on the progression of CCD^27^. Additionally, polymerase chain reaction (PCR) for *T. cruzi* has proven to be a valuable tool, enhancing the sensitivity of parasite detection in CCD and becoming a key biomarker for treatment response assessment^43^.

Beginning in the 2000s, guidelines and consensus documents from some endemic countries recommended the use of BZN or NFX in adults with CCD. However, since 2018, official guidelines have established criteria for trypanocidal treatment, including one from the Pan American Health Organization (PAHO) and two others from the Brazilian and Argentine Ministries of Health ^2^ ^,^ ^44^ ^,^ ^45^. Despite being based on low-quality evidence, these guidelines suggest that, in most cases, the balance between benefits and risks is favorable. Considering the potential benefits of slowing disease progression and reducing transmissibility, etiological treatment is recommended for individuals with CCD who have no specific organ damage or mild cardiomyopathy, are aged <50 years, and have no severe comorbidities. For patients aged >50, the benefits are uncertain, and treatment should be assessed on a case-by-case basis.

Official guidelines strongly recommend trypanocidal treatment for children and adolescents with chronic infections due to its greater efficacy and better tolerability. They also highlight the importance of treating women of childbearing age to prevent vertical transmission during future pregnancies. Conversely, they advise against treatment during pregnancy, especially in the first trimester, and in patients with chronic infections and advanced organ damage, particularly severe cardiomyopathy, where the potential benefits are uncertain and the risk of adverse effects is high. This conditional recommendation was supported by moderate-quality evidence. In addition, these studies underscore the need for the close monitoring of ADRs with dose adjustments or treatment interruptions in cases of severe events (Table 1).

**TABLE 1:** Comparative Table: Etiological Treatment for Chronic Chagas Disease in Adults.

Aspect	Brazilian PCDT (CONITEC, 2018)	PAHO/WHO Guidelines (2018)
Target population	Adults with chronic Chagas disease, especially those aged <50 years, with good life expectancy and no serious comorbidities.	Adults with chronic Chagas disease without specific organ damage.
General recommendation	Treatment may be considered. Stronger recommendation for individuals aged <50 years, with a favorable clinical profile.	Conditional recommendation in favor of treatment. Benefits likely outweigh harms in most cases.
Patients >50 years	Treatment is not routinely recommended due to uncertain benefit. It may be recommended on a case-by-case basis.	No explicit cutoff age. Decisions should consider risks and individual factors.
Patients with organ damage (cardiac/digestive)	Not a routine indication. Requires specialized assessment.	Conditional recommendation against treatment in patients with established organ damage. Evidence of moderate quality.
Women of childbearing age	Treatment is recommended before pregnancy to prevent congenital transmission.	Strong recommendation for treatment to reduce the risk of vertical transmission.
Children/adolescents	Strong recommendation for treatment due to better efficacy and tolerance.	Strong recommendation for etiological treatment in all pediatric patients.
Preferred drugs	Benznidazole (first-line), Nifurtimox (alternative).	Benznidazole or Nifurtimox, with no preference stated. Choice depends on availability and tolerance.
Evidence quality	Based on GRADE. Evidence considered low to moderate, but favorable in selected groups.	GRADE-based. Evidence rated as low (for adults without organ damage) and moderate (for women and children).
Monitoring and adverse effects	Requires clinical monitoring. Adverse effects may lead to dose adjustment or discontinuation.	Similar emphasis on safety monitoring and management of adverse drug reactions.
Implementation emphasis	Treatment should be discussed with the patient; benefits, risks, and alternatives explained.	Emphasis on shared decision-making and expanding access in primary healthcare settings.

## CURE CRITERIA

Clinically, *T. cruzi* factors such as low parasitemia, long-term persistence of positive serological tests, and slow clinical progression of the disease make it difficult for adults to experience the effectiveness of trypanocidal treatment for CCD^46^. Thus, the need for long-term follow-up of treated patients presents a significant limitation in evaluating cures in clinical trials. Another relevant issue regarding the specific treatment of CCD is the definition of cure criteria. There are three complementary criteria: parasitological, serological, and clinical^46^. Parasitological criterion refers to the sustained absence of the parasite or its DNA in peripheral blood samples. Serological criteria are based on seroconversion or seronegativization, defined as the loss of reactivity in conventional serological tests that were positive before treatment. The clinical criteria consider disease progression, such as the evolution from the indeterminate form to the cardiac form, or progression across the clinical stages of cardiomyopathy. In other words, specific treatment is expected to influence disease progression by preventing or delaying the development of defined clinical forms in patients with the indeterminate form or by halting or slowing the worsening of cardiomyopathy in more advanced stages.

Owing to the extended timeframe required for clinical and serological outcomes, clinical trials typically adopt parasitological criteria as their primary endpoint^47^. This indicates that patients with positive PCR results before treatment should exhibit sustained negative PCR results after treatment. In clinical trials of CD, the follow-up period using PCR after treatment has not yet reached a single well-defined consensus. However, some recent guidelines and practices suggest a minimum period of 12 months with serial assessments during this time, which may be extended to 24-36 months in more robust studies^48^. Long-term observational studies spanning approximately two decades have shown variable serological responses in treated patients, with some achieving seroconversion over extended periods^49^. Similarly, assessing the clinical effect of treatment on disease progression requires prolonged observation periods, as demonstrated in studies with follow-up periods extending beyond 20 years^50^.

## ASSESSMENT OF CURE

Systematic reviews are essential for assessing trypanocidal therapies and their therapeutic outcomes in CCD. By gathering, critically appraising, and synthesizing available data from multiple clinical and observational studies, these reviews provide a comprehensive overview of the efficacy and safety of therapeutic interventions such as the use of BZN and NFX^51^. In the context of CCD, where clinical outcomes may vary significantly between studies owing to population heterogeneity, different inclusion criteria, clinical forms, and methodologies employed, systematic reviews help identify consistent patterns of benefit, reduce individual biases, and improve the precision of effect estimates. They also enable the identification of knowledge gaps, such as the lack of robust data in specific subgroups, thereby guiding future research. Furthermore, when combined with meta-analyses, systematic reviews offer quantitative evidence that can inform clinical decisions and public health policies, strengthening recommendations for etiological treatment, even in the presence of methodological limitations in primary studies. As such, they represent an essential tool to support the expansion of treatment access and the standardization of therapeutic approaches for CCD.

Two systematic reviews on the use of BZN in the treatment of CCD were conducted in 2007^52^ and 2009^53^. These reviews identified only four and nine studies, respectively, that met the predefined eligibility criteria. The first review included only randomized trials, whereas the second included both randomized and observational studies. At the time, the available data indicated that treatment efficacy for CCD was uncertain. This uncertainty was more pronounced in symptomatic individuals and those aged >50 years, where the risk-benefit balance appeared unfavorable. Although some data suggest a potential benefit, it might have been marginal, and most information on treatment during the late chronic phase came from non-randomized studies. The results varied depending on disease stage, treatment duration and dosage, patient age, and geographical origin. In addition, the use of heterogeneous evaluation protocols involving different endpoints has contributed to the ongoing controversy regarding the specific treatment for CCD.

In 2021, a systematic review of clinical and observational studies on antiparasitic treatment highlighted the diversity of approaches used in the clinical management of CCD and the significant variations in how clinical studies are conducted^54^. However, a recent systematic review and meta-analysis published in 2025 pointed to a paradigm shift in the treatment of CCD, indicating positive clinical outcomes associated with etiological therapy^55^. Consistent evidence shows that antiparasitic treatment significantly reduces the risk of electrocardiographic abnormalities, disease progression, cardiovascular death, and overall mortality in patients with CCD, including those with mild cardiomyopathy. Although the quality of evidence ranges from low to intermediate and considerable heterogeneity exists among the studies analyzed, the potential benefits of treatment are substantial^55^. 

## THERAPEUTICS

According to the PAHO/World Health Organization (WHO) requirements, a trypanocidal drug should meet the following criteria: achieve parasitological cure in both acute and chronic cases, be effective as a single daily dose or in a few doses, have low cost, have minimal side effects, not induce resistance, and not require a hospital setting for use^56^. Although BZN and NFX do not fully meet the criteria established by PAHO/WHO, they are currently available for the treatment of CD.

BZN is available in 100 mg and 50 mg tablets, as well as a 12.5 mg pediatric formulation, all administered orally in two daily doses. The doses by age/weight are 5-7 mg/kg/day for adults and 5-10 mg/kg/day for children. The standard treatment duration is 60 days; however, in some cases (depending on the patient’s weight), it may be extended to 80 days^44^. NFX is available as 30 mg and 120 mg tablets and is administered orally at two to three doses per day. The dosage by age/weight is 8-10 mg/kg/day for adults and 10-20 mg/kg/day for children. The treatment duration was 60 days^44^ (Table 2).

**TABLE 2:** Comparative Table: Benznidazole vs Nifurtimox.

Criteria	Benznidazole	Nifurtimox
Pharmaceutical Formulation	100 mg and 12.5 mg tablets (oral use)	120 mg and 30 mg tablets (oral use)
Dosage - Adults	5-7 mg/kg/day (twice daily, orally)	8-10 mg/kg/day (three to four times/day, orally)
Dosage - Children	5-10 mg/kg/day (twice daily, orally)	10-20 mg/kg/day (three to four times/day, orally)
Treatment Duration	60 days (may vary; up to 80 days)	60 days
Main Adverse Events	Nausea, vomiting, allergic dermatitis, and peripheral neuropathy	Anorexia, weight loss, vomiting, abdominal pain, and insomnia
Main Contraindications	Pregnancy, severe neurological diseases, and severe liver disease	Pregnancy, severe neurological or psychiatric disorders, and severe liver disease

Treatment is strongly recommended during the acute phase, congenital infections, and in children and adolescents. In adults in the late chronic phase, the decision should be individualized, considering age (<50 years), absence of advanced Chagas cardiomyopathy, treatment adherence and tolerance, and the risk-benefit balance regarding potential adverse effects. Treatment is contraindicated during pregnancy (especially in the first trimester), in cases of severe hepatic or renal failure, and in cases of uncontrolled neurological or psychiatric disorders^44^.

## MANAGEMENT OF ADVERSE EVENTS

ADRs occur in approximately 30-50% of adult patients treated with trypanocidal drugs. Therefore, it is crucial to educate patients about the importance of completing treatment and inform them about the potential ADRs, which should be promptly reported to healthcare providers. When ADRs occur, the attending physician may manage them symptomatically using medications or, if necessary, discontinue treatment temporarily or permanently^46^. Within the Primary Healthcare framework, community health workers play a key role in patient monitoring and support, including in the early detection of ADRs. Their involvement is essential for ensuring treatment continuity, particularly in small municipalities with limited healthcare resources^57^.

The most common adverse events associated with BZN include dermatological reactions, primarily allergic dermatitis; neurological effects such as peripheral neuropathy; gastrointestinal symptoms, including anorexia, nausea, and vomiting; hepatic abnormalities such as elevated transaminase levels; and hematological issues such as leukopenia. Among these, gastrointestinal symptoms tended to occur in the first week of treatment, dermatitis occurred between the first and second weeks, and neuropathy generally occurred in the final weeks of treatment^58^ (Table 3). Regarding NFX, the primary adverse events include gastrointestinal symptoms, such as nausea, vomiting, abdominal pain, and anorexia, often accompanied by clinically significant weight loss; neurological effects, including irritability, insomnia, headache, paresthesia, and dizziness; psychiatric manifestations, such as behavioral disturbances, nightmares, depression, and both passive and active suicidal ideation; and dermatological reactions, such as skin rashes and pruritus. Similar to BZN, NFX showed a temporal incidence pattern of ADR. A peak in ADR was observed between the second and fourth weeks of treatment, with a high frequency of vomiting, weight loss, skin reactions, and neuropsychiatric symptoms^59^ (Table 4).

**TABLE 3:** Temporal Distribution of Main Adverse Events during Benznidazole Treatment

Treatment Phase	Common Adverse Events (AEs)
Week 1 (Initiation Phase)	- Nausea
	- Mild skin rash
	- Headache
	- Abdominal discomfort
Weeks 2-4 (Peak Incidence)	- Generalized dermatitis (rash and pruritus)
	- Allergic reactions (urticaria and angioedema)
	- Neuropathy (paresthesia and peripheral neuritis)
	- Leukopenia (rare but serious)
After Week 4 (Late Phase)	- Neuropathy (paresthesia and peripheral neuritis)
	- Symptoms may worsen or become intolerable
	- May require dose reduction, temporary suspension, or permanent discontinuation
	- Continued vigilance for delayed-onset neuropathy

**TABLE 4:** Temporal Distribution of Main Adverse Events during Nifurtimox Treatment.

Treatment Phase	Common Adverse Events (AEs)
Week 1 (Initiation Phase)	- Nausea
	- Anorexia
	- Headache
	- Abdominal pain
	- Fatigue
Weeks 2-4 (Peak Incidence)	- Vomiting
	- Weight loss
	- Skin reactions (rash and pruritus)
	- Neuropsychiatric symptoms (irritability, insomnia, confusion, and paresthesia)
	- Elevated liver enzymes (rare)
After Week 4 (Late Phase)	- Symptoms may stabilize or subside
	- Persistent or severe AEs may require dose adjustment or treatment discontinuation

Owing to the high incidence of ADRs, approximately 30% of patients permanently discontinue treatment^51^. Therefore, early detection and proper management of ADRs are critical to ensuring the successful completion of etiological treatment for CD. Monitoring treatment with a focus on ADRs and safeguarding patient safety requires the timely identification of these reactions^60^. To support this, adherence to a standardized clinical protocol for therapeutic management is recommended. A clinical protocol is a structured document that compiles guidelines based on scientific evidence and aims to standardize patient care, ensuring the quality, safety, and efficiency of a clinical procedure. In the specific case of trypanocidal drug treatment, a structured and officially recommended clinical protocol is lacking. However, some clinical trials have adopted protocols that involve periodic clinical and laboratory assessments. These protocols typically recommend that, before starting treatment, blood tests, such as complete blood count and transaminases, urea, and creatinine levels, should be performed, along with dermatological and neurological evaluations**.** For women of childbearing age, pregnancy testing is mandatory before treatment initiation and effective contraception must be ensured throughout the treatment course, as both BZN and NFX are contraindicated during pregnancy^61^. On day 7, a dermatological evaluation should be performed, and in case of a skin rash, the possibility of discontinuing treatment should be considered, along with additional blood tests. On day 15, dermatological and hematological evaluations are recommended. In cases of leukopenia, the potential need to discontinue treatment and perform further testing should be assessed. On day 30, hematological and neurological evaluations are conducted to detect signs of peripheral neuropathy that may require treatment interruption. Clinical and laboratory evaluations are repeated on days 45 and 60, and additional blood tests are performed as needed (Table 5)^62^.

**TABLE 5:** Example of a protocol for managing adverse reactions to benznidazole

Timepoint	Visit day 0	Visit day 7	Visit day 15	Visit day 30	Visit day 45	Visit day 60
Blood tests	X	X	X	X	X	
Dermatological evaluation		X	X	X		
Hematological evaluation			X	X	X	
Neurological evaluation				X	X	X
Application of the ADR risk score	X					

According to the WHO, ADRs can be classified as mild, moderate, or severe, and the assessment of causality is categorized as certain, probable, possible, unlikely, conditional, or unclassifiable^63^ ^,^ ^64^. When a symptom or sign is recognized as an ADR to a trypanocidal drug, clinical management involves symptomatic treatment without interrupting therapy in mild cases, temporary suspension in moderate cases, and permanent discontinuation in severe cases. During the first week of treatment, when gastrointestinal symptoms are more frequent, antiemetics, prokinetics, antisecretory agents, and antispasmodics are commonly administered. In specific cases of dermatitis, depending on the severity of the condition (whether mild or moderate), management may include the use of moisturizing creams, antihistamines, and/or corticosteroids. Neuropathy management requires definitive discontinuation of trypanocidal drugs and the initiation of neuromodulatory medications. Regarding the main laboratory alterations, the management of leukopenia is based on the absolute neutrophil count: in mild cases (1,000-1,499), observation with weekly monitoring is recommended; in moderate cases (500-999), temporary suspension is indicated; and in severe cases (<500), the drug must be discontinued^65^. Additional discontinuation criteria included the elevation of transaminases (alanine aminotransferase and aspartate aminotransferase) greater than three times the upper limit of normal, which requires treatment interruption and close monitoring until normalization. Notably, the use of a risk score may help predict the likelihood of ADRs, enabling closer monitoring of patients considered at higher risk, typically younger Caucasians and highly educated women^66^.

## PUBLIC POLICIES

Since the 1980s, national CD control programs have been implemented in endemic countries, with a primary focus on eliminating vector transmission of *Triatoma infestans*. Regional initiatives supported by the PAHO and WHO, such as the Southern Cone Initiative against CD, have coordinated intergovernmental alliances for control, surveillance, and treatment^67^. CD, already recognized by the WHO as a Neglected Tropical Disease (NTD), was further highlighted when World Chagas Disease Day (April 14) was approved by the World Health Assembly in May 2019, with the first celebration held in 2020 to raise visibility^68^. In 2021, the WHO launched the 2021-2030 global strategy against NTDs, which included specific goals to eliminate the vectorial and vertical transmission of CD^69^. Recently, public policies have increasingly incorporated the PAHO/WHO recommendations, which support etiological treatment not only for children and adolescents but also for adults with the indeterminate chronic form or mild cardiomyopathy and for women of childbearing age as a strategy to prevent vertical transmission^4^.

Although access to diagnosis and treatment remains limited, progress has been made in the distribution of rapid diagnostic tests, which facilitate early diagnosis and expand the number of patients eligible for treatment. This has been complemented by the free distribution of BZN and NFX and the training of healthcare professionals^70^. Non-governmental organizations (NGOs) and civil society play key roles in both endemic and urban areas. Organizations, such as the Médecins Sans Frontières (MSF), Drugs for Neglected Diseases initiative (DNDi), and Mundo Sano Foundation, support projects aimed at improving access to diagnosis and treatment, conducting clinical research, developing medicines, and promoting health education^71^ ^-^ ^73^ Over the last few decades, patient advocacy and activism have led to the formation of local and regional associations among people affected by CD, both in endemic and non-endemic countries. This mobilization culminated in the creation of the International Federation of Associations of People Affected by Chagas Disease, which represents patients globally, promotes political engagement, raises awareness, and fights stigma, supporting regional patient associations and social movements to defend the right to diagnosis and etiological treatment^74^.

DNDi, in partnership with institutions such as Fiocruz, has led to the development of new drugs, including pediatric formulations and the production of more accessible and sensitive diagnostic tests^75^. NGOs and universities collaborate on projects such as IntegraChagas, the Chagas Platform, and networks, such as NHEPACHA, focusing on trypanocidal treatment and cure monitoring^76^ ^-^ ^78^. NGOs also participate in international forums to influence global health policies, secure funding, and ensure that CD are included on international cooperative agendas. They promote public-private partnerships and the integration of CD care into primary healthcare systems. Organizations, such as the MSF and other local NGOs, have facilitated screening and treatment campaigns in hard-to-reach areas, including rural regions in Bolivia, Colombia, Guatemala, and Brazil’s Amazon region. Through networks, such as the Chagas Platform, NGOs collaborate with governments and universities to train health teams, integrate CD care into primary health units, implement patient-centered care models, and produce educational materials and simplified clinical protocols, including ADR management. NGOs and research centers have also helped develop active pharmacovigilance systems, which are essential for effective ADR management and improved treatment adherence.

## FUTURE PERSPECTIVES

Despite progress in public health policies, the etiological treatment of CD still faces major limitations, especially regarding tolerability and patient adherence to BZN and NFX. Several strategies are being developed to improve the efficacy, reduce adverse events, and expand access to treatment^79^. Recent clinical studies, such as the BENDITA trial, have evaluated shorter treatment regimens with BZN (such as 2 weeks instead of 60 days) or reduced doses. These promising results suggest that shorter or lower-dose regimens may maintain parasitological efficacy while reducing the incidence of ADRs, thus improving adherence^40^. Ongoing studies aim to validate treatment regimens tailored by age group, clinical form, and risk-benefit profile.

Another approach involves drug repositioning and combination therapies with BZN, focusing on the repurposing of drugs already approved for other diseases to achieve synergistic effects and reduce toxicity. Although several promising candidates have been evaluated in clinical trials, the results have been disappointing. Examples include ravuconazole (E1224)^38^, an antifungal prodrug that showed only transient parasitological clearance with no sustained efficacy in phase 2 trials; fosravuconazole^40^, another ravuconazole prodrug that failed to demonstrate superiority over BZN monotherapy in the BENDITA trial; and posaconazole, which was evaluated in some trials (CHAGASAZOL^35^ and STOP-CHAGAS^37^) but showed inferior efficacy compared with BZN, with high rates of treatment failure. Other compounds, such as amiodarone^80^, have been explored in experimental models, and disulfiram^62^ has been investigated in combination with BZN chemotherapy for CD. Anti-inflammatory and immunomodulatory agents have also been studied as potential adjuvants to mitigate myocardial damage. The overall goal of combination therapy is to improve parasitological efficacy, shorten treatment duration, and minimize ADRs, although this strategy remains in the experimental or early clinical trial stages^81^. 

Conversely, efforts to develop new drugs with novel therapeutic targets are advancing, with several innovative molecules being studied for mechanisms distinct from those of the traditional nitro derivatives, BZN and NFX, with the aim of overcoming parasite resistance and improving safety profiles. Among the most promising candidates is LXE408, developed by Novartis, which inhibits an essential *T. cruzi* enzyme and is currently undergoing clinical evaluation (CLXE408B12201)^82^. Fexinidazole, another drug already approved for the African trypanosomiasis, demonstrated high efficacy in parasite clearance compared with a placebo in a clinical trial conducted in Bolivia; however, the study was halted owing to safety concerns^83^. Other compounds, including nitroimidazoles, cruzipain inhibitors, proteasome inhibitors, redox enzyme blockers, benzoxazole analogs, and drugs with alternative mechanisms of action, are in preclinical development stages^84^ ^-^ ^87^. The search for curative biomarkers is critical for the success of these studies, as the clinical outcomes of CCD can take many years to manifest.

Although still in the early phases, research on therapeutic vaccines aimed at modulating the immune response in already infected patients and prophylactic vaccines to prevent infection is advancing^88^. Some of the vaccines under investigation use recombinant antigens, such as Tc24 and trans-sialidase, combined with immunological adjuvants. Experimental mouse models have demonstrated reduced parasitic loads and protection against cardiac damage in mice^89^. In the future, vaccines may be used in combination with BZN to increase therapeutic response rates and prevent the progression of cardiomyopathy.

These innovations point to a more promising future for CCD management; however, regulatory, logistical, and financial challenges still need to be overcome for new therapeutic approaches to translate into concrete benefits for millions of affected patients, particularly those most vulnerable in endemic regions.

## CONCLUSION

Trypanocidal therapy for CCD is evolving, marked by significant challenges. Despite the formal availability of BZN and NFX, their use is limited because of the frequent occurrence of ADRs, which affect treatment adherence and completion. Although etiological treatment is recommended by international guidelines, it remains restricted to specific groups, such as children, adolescents, young adults with indeterminate forms or mild cardiomyopathy, and women of childbearing age, reflecting a cautious approach based on risk-benefit considerations and the limited availability of high-quality evidence.

Proper management of ADRs, together with active pharmacovigilance and clinical monitoring, is essential to ensure continuity of treatment. Primary health care and multidisciplinary teams, including community health workers, play strategic roles, particularly in areas with limited infrastructure. Shorter treatments, reduced dosages, and combination therapies offer a promising horizon, although their implementation depends on the outcomes of ongoing clinical trials. The development of new drugs with innovative therapeutic targets and research on therapeutic and prophylactic vaccines could expand treatment options in the future.

Although treatment is officially available, access remains constrained by structural, informational, and cultural barriers. Contributing factors include insufficient awareness of chronic infections among both patients and healthcare professionals, delayed diagnosis, apprehension regarding potential drug-related adverse effects, and the absence of systematic pharmacovigilance in many regions. It is estimated that fewer than 10%, and often around 1% or less, of individuals infected with *T. cruzi* in endemic countries actually receive trypanocidal treatment. Finally, the critical roles of public policies, civil society organizations, and collaborative research networks must be highlighted. These actors help expand access to diagnoses and treatments, promote health education, and advocate for the rights of affected individuals. Overcoming structural, regulatory, and financial barriers is essential for transforming scientific advances into real benefits for millions of patients with CD.

## Data Availability

Data usage not reported, research data not used.
